# Supplementary material for: Characterization and Experimental Use of Multiple Myeloma Bone Marrow Endothelial Cells and Progenitors
Source: Int J Mol Sci. 2024 Nov 9;25(22):12047. doi: 10.3390/ijms252212047 (PMC11594118; doi:10.3390/ijms252212047)
Supplement: Supplementary file 1 [file ijms-25-12047-s001.zip › Supplementary Figs and Table S1.pdf]

Supplementary Materials

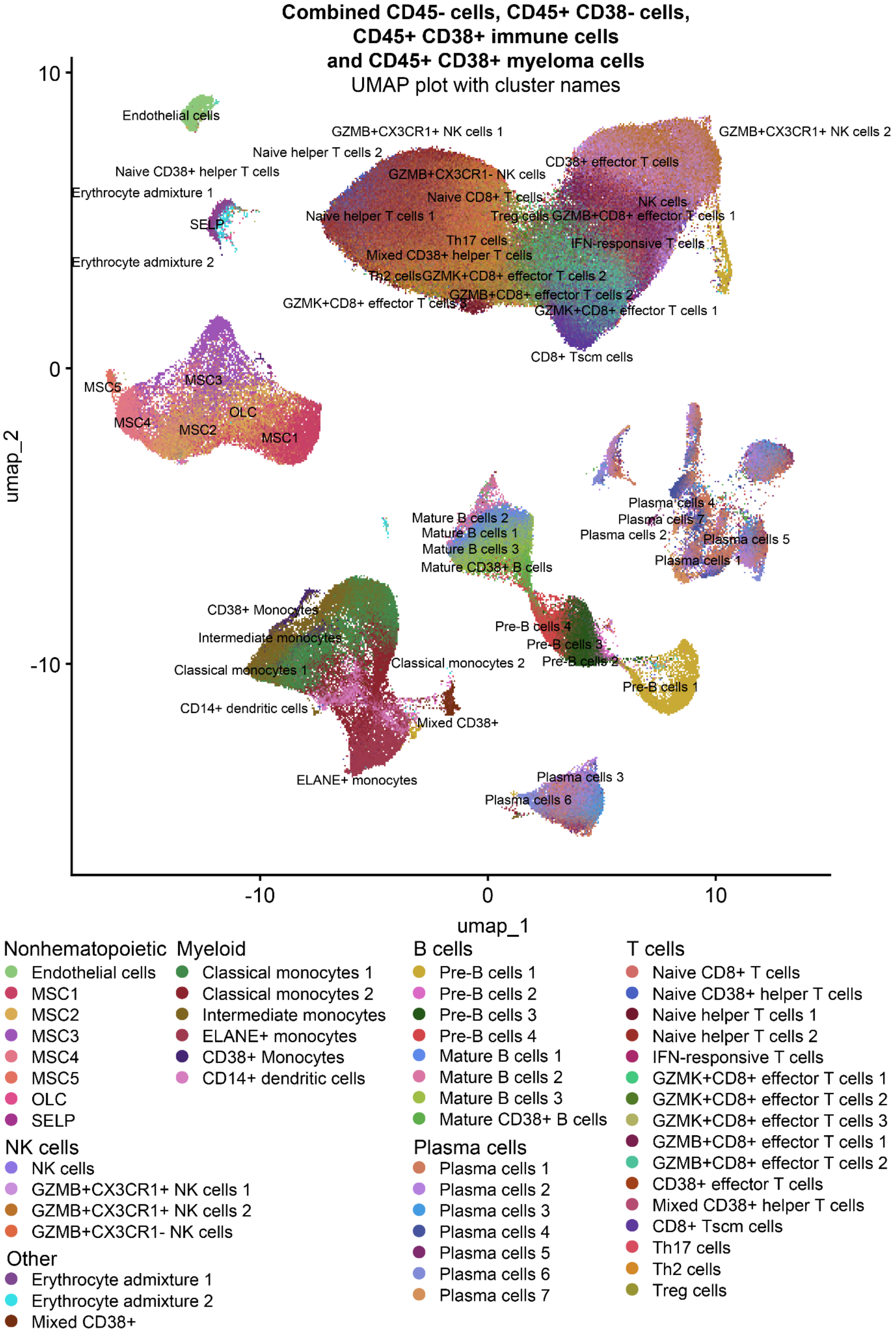

Figure S1. UMAP projection illustrating the full cluster annotation of the merged datasets.

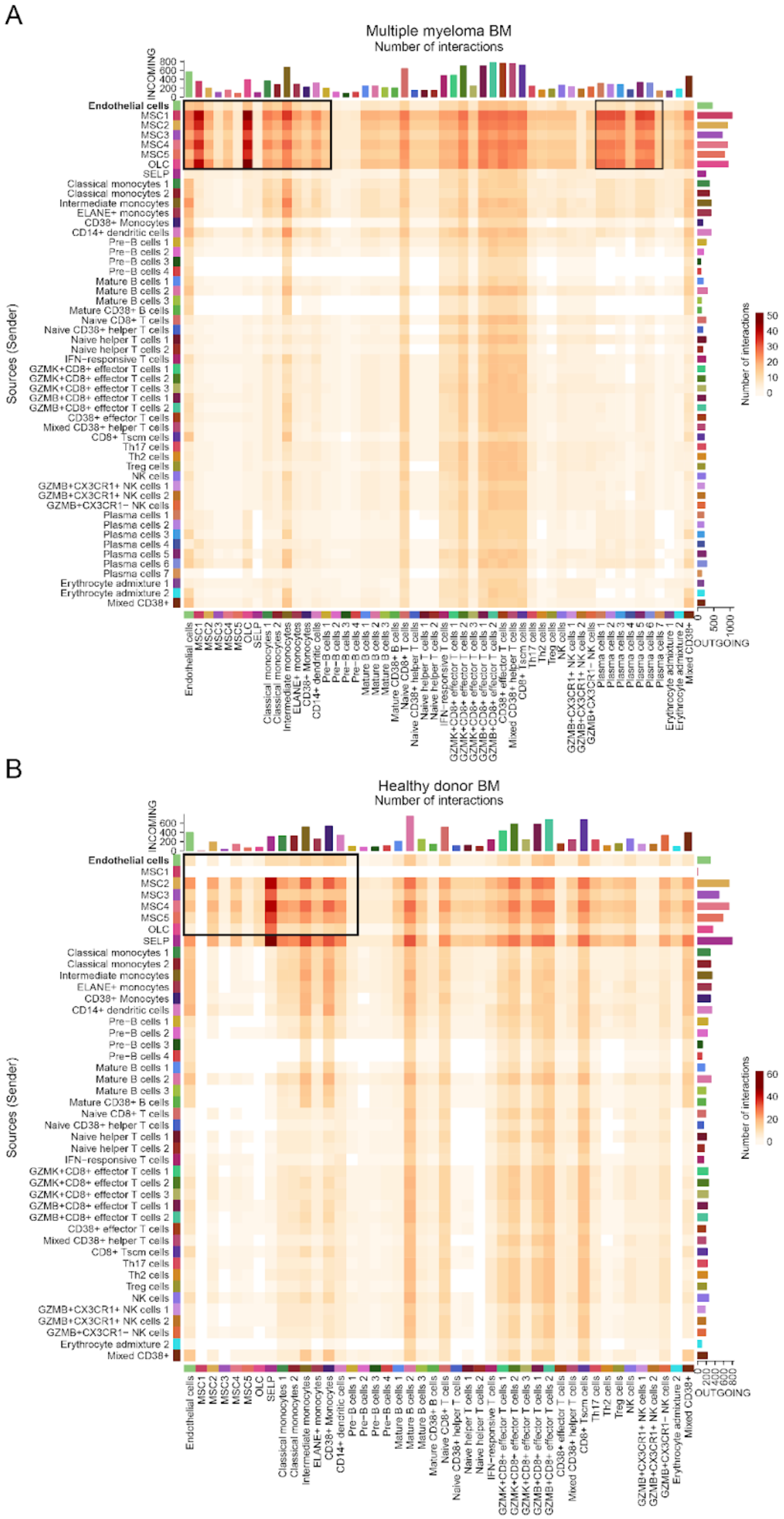

Figure S2. Summarized incoming and outgoing cluster-cluster interactions in MM **(A)** and HD **(B)** BM. Heatmaps depict the number of predicted cell-cell interactions in the bone marrow of multiple myeloma patients, with interactions classified as incoming (afferent, vertical axis) and outgoing (efferent, horizontal axis) signaling. The intensity of the color represents the number of interactions, with darker colors indicating more interactions.

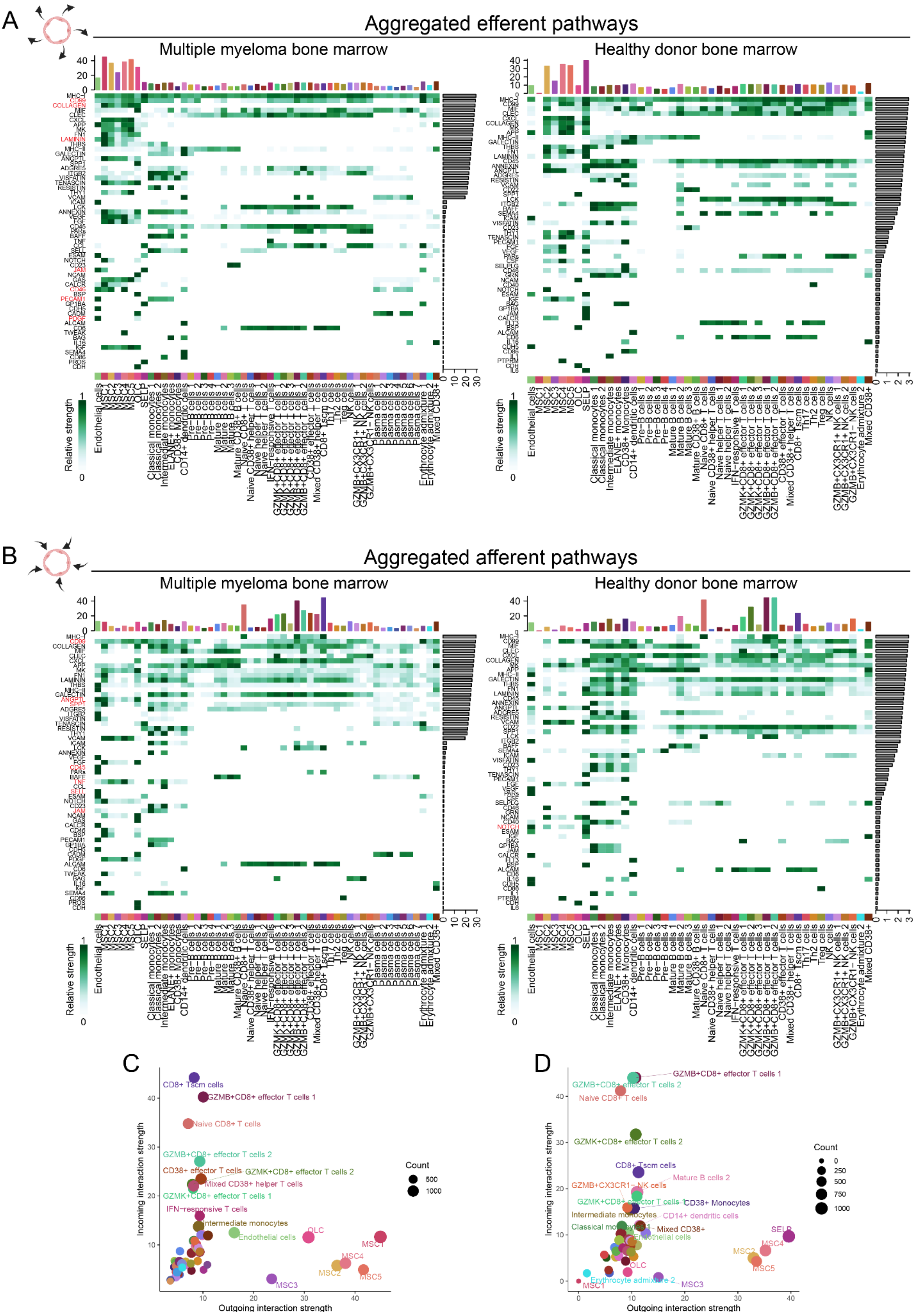
A
Aggregated efferent pathways
Multiple myeloma bone marrow
Healthy donor bone marrow
Relative strength
B
Aggregated afferent pathways
Multiple myeloma bone marrow
Healthy donor bone marrow
Relative strength
C
Incoming interaction strength
Outgoing interaction strength
Count
CD8+ Tscm cells
GZMB+CD8+ effector T cells 1
Naive CD8+ T cells
GZMB+CD8+ effector T cells 2
CD38+ effector T cells
GZMK+CD8+ effector T cells 2
Mixed CD38+ helper T cells
GZMK+CD8+ effector T cells 1
IFN−responsive T cells
Intermediate monocytes
Endothelial cells
OLC
MSC1
MSC4
MSC2
MSC5
MSC3
D
Incoming interaction strength
Outgoing interaction strength
Count
GZMB+CD8+ effector T cells 1
GZMB+CD8+ effector T cells 2
Naive CD8+ T cells
GZMK+CD8+ effector T cells 2
CD8+ Tscm cells
Mature B cells 2
GZMB+CX3CR1−NK cells
GZMK+CD8+ effector T cells 1
CD38+ Monocytes
Intermediate monocytes
CD14+ dendritic cells
Classical monocytes 1
Mixed CD38+
Endothelial cells
SELP
MSC4
MSC2
MSC5
OLC
Erythrocyte admixture 2
MSC1
MSC3

Figure S3. Aggregated efferent and afferent signaling pathways in MM and HD BM. Heatmaps depicting aggregated efferent **(A)** and afferent **(B)** signaling pathways which are predicted to be present in ECs from MM bone marrow (left) and HD bone marrow (right). The strength of interactions is displayed, with darker shades of green representing stronger interactions. The y-axis lists aggregated pathway names. Bar plots at the top represent the number of interactions per cell type, with relative strength shown on the x-axis. Summary of interaction strength for each cluster in MM bone marrow **(C)** and HD bone marrow **(D)**.

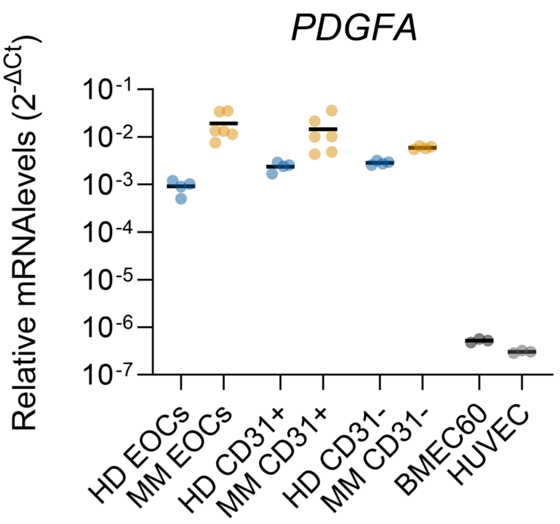

HD EOCs vs. HD CD31+	$p = 0.02$
MM EOCs vs. MM CD31+	$p = 0.98$
HD CD31+ vs. HD CD31-	$p = 0.54$
MM CD31+ vs. MM CD31-	$p = 0.51$
HD EOCs vs. MM EOCs	$p = 0.06$
HD CD31+ vs. MM CD31+	$p = 0.24$

Figure S4. *PDGFA* expression levels in indicated samples. Statistical significance was assessed using Brown-Forsythe and Welch ANOVA tests with Dunnett's T3 correction for multiple comparisons. HD EOCs n = 4, MM EOCs n = 6, HD CD31+ n = 4, MM CD31+ n = 6, HD and MM CD31- n = 4, cell lines n = 3. Horizontal line indicates mean.

Table S1. Oligonucleotide sequences used for Real-Time qPCR.

Gene symbol	Oligonucleotide sequences
PTPRC	F: ACCACAAGTTTACTAACGCAAGT
	R: TTTGAGGGGGATTCCAGGTAAT
PECAM1	F: AACAGTGTTGACATGAAGAGCC
	R: TGTAAAACAGCACGTCATCCTT
CD34	F: CTACAACACCTAGTACCCTTGGA
	R: GGTGAACACTGTGCTGATTACA
KDR	F: GGCCCAATAATCAGAGTGGCA
	R: CCAGTGTCATTTCCGATCACTTT
PROM1	F: AGTCGGAAACTGGCAGATAGC
	R: GGTAGTGTTGTACTGGGCCAAT
CDH5	F: TTGGAACCAGATGCACATTGAT
	R: TCTTGCGACTCACGCTTGAC
STAB2	F: GTGCCCGGATGGTTACACC
	R: CTTCCTACAAATATGGCGGCAT
VCAM1	F: GGGAAGATGGTCGTGATCCTT
	R: TCTGGGGTGGTCTCGATTTTA
APLNR	F: CTCTGGACCGTGTTTCGGAG
	R: GGTACGTGTAGGTAGCCCACA
EFNB2	F: TATGCAGAACTGCGATTTCCAA
	R: TGGGTATAGTACCAGTCCTTGTC
SOX17	F: GTGGACCGCACGGAATTTG
	R: GGAGATTCACACCGGAGTCA
FLT1	F: TTTGCCTGAAATGGTGAGTAAGG
	R: TGGTTTGCTTGAGCTGTGTTC
PDGFA	F: GCAAGACCAGGACGGTCATTT
	R: GGCACTTGACACTGCTCGT
VIM	F: GACGCCATCAACACCGAGTT
	R: CTTTGTCGTTGGTTAGCTGGT
STC1	F: GTGGCGGCTCAAAACTCAG
	R: GTGGAGCACCTCCGAATGG
YWHAZ	F: AGGAGATTACTACCGTTACTTGGC
	R: AGCTTCTTGGTATGCTTGTTGTG
ACTB	F: CATGTACGTTGCTATCCAGGC
	R: CTCCTTAATGTCACGCACGAT
RNA18S	F: CGTCTGCCCTATCAACTTTG
	R: TGCCTTCCTTGGATGTGGTAG
